# Epigenetic dysregulation: a novel pathway of oncogenesis in pediatric brain tumors

**DOI:** 10.1007/s00401-014-1325-8

**Published:** 2014-07-31

**Authors:** Adam M. Fontebasso, Tenzin Gayden, Hamid Nikbakht, Michael Neirinck, Simon Papillon-Cavanagh, Jacek Majewski, Nada Jabado

**Affiliations:** 1 Division of Experimental Medicine, Montreal Children’s Hospital, McGill University and McGill University Health Centre, 4060 Ste Catherine West, PT239, Montreal, QC H3Z 2Z3 Canada; 2 Department of Human Genetics, McGill University, Montreal, QC Canada

## Abstract

A remarkably large number of “epigenetic regulators” have been recently identified to be altered in cancers and a rapidly expanding body of literature points to “epigenetic addiction” (an aberrant epigenetic state to which a tumor is addicted) as a new previously unsuspected mechanism of oncogenesis. Although mutations are also found in canonical signaling pathway genes, we and others identified chromatin-associated proteins to be more commonly altered by somatic alterations than any other class of oncoprotein in several subgroups of childhood high-grade brain tumors. Furthermore, as these childhood malignancies carry fewer non-synonymous somatic mutations per case in contrast to most adult cancers, these mutations are likely drivers in these tumors. Herein, we will use as examples of this novel hallmark of oncogenesis high-grade astrocytomas, including glioblastoma, and a subgroup of embryonal tumors, embryonal tumor with multilayered rosettes (ETMR) to describe the novel molecular defects uncovered in these deadly tumors. We will further discuss evidence for their profound effects on the epigenome. The relative genetic simplicity of these tumors promises general insights into how mutations in the chromatin machinery modify downstream epigenetic signatures to drive transformation, and how to target this plastic genetic/epigenetic interface.

## Current challenges in subgroups of high-grade pediatric brain tumors

**Fig. 1:**
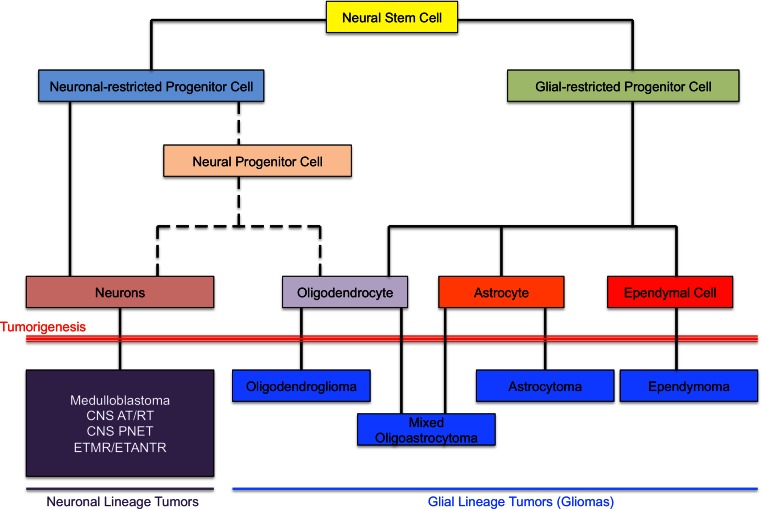
Central nervous system cellular development and tumorigenesis. Graphic depiction showing differentiation of neural stem cells into neuronal and glial differentiation pathways and subsequent tumorigenesis from presumed cells of origin. *CNS* Central nervous system, *PNET* primitive neuroectodermal tumor, *AT/RT* atypical teratoid/rhabdoid tumor, *ETMR* embryonal tumor with multilayered rosettes, *ETANTR* embryonal tumor with abundant neuropil and true rosettes

According to the most recent reports published in 2014, one in every 285 children will be diagnosed with cancer before the age of 20 years [73]. Overall survival has improved substantially over the last three decades for specific cancer types, mainly childhood leukemia, partly based on better stratification of patients using molecular tools. In contrast, a subset of tumors remains incurable today and includes subgroups of brain tumors, a leading cause of cancer-related morbidity and mortality in the pediatric years. Several impediments to effective treatment exist and hamper the design and outcome of needed novel clinical trials. Diagnosis still relies mainly on standard pathology that characterizes tumors according to the World Health Organization (WHO) classification. Tumors are classified according to their presumed cell of origin and then are further divided into distinct histological grades, ranging from WHO grade I to WHO grade IV based on cytologic and histologic features (Fig. 1). Moreover, these tumors are often studied and treated as if they were analogous to adult tumors. However, pediatric brain tumors of most pathological types appear to harbor unique molecular alterations compared to these very same tumors occurring in the adult years, although under the microscope they are indistinguishable [18, 20, 50, 56, 63, 66]. Indeed, we and others have shown that they represent unique molecular entities and may require distinct therapeutic approaches [1, 2, 11, 18, 23, 26, 27, 30, 31, 50, 58, 69]. Supporting this is the predilection for pediatric and adult tumors to occur in different brain regions, with tumors arising in particular areas harboring distinct genetic alterations [63, 66, 75]. Last but not least, in the context of high-grade tumors, recent work enabled by next-generation sequencing (NGS) technologies has begun to point us in the direction of the epigenome as a major driver of cancer development [63, 75]. Recent findings from several groups including ours point to the epigenome as a previously under-appreciated hallmark of oncogenesis that drives several groups of intractable high-grade pediatric brain tumors. These mutations directly affect histone genes, the core component of chromatin, or post-translational modifications affecting specific residues within the histone tail as well as enzymes mediating DNA methylation. Consequently, better stratification of patients based on tumor biology, improved identification of relevant therapeutic targets, and the design of experimental models to test compounds affecting specific genetic/molecular drivers are essential for therapeutic breakthroughs in these deadly diseases. Moreover, epigenetic alterations observed in high-grade pediatric brain tumors result in a previously unforeseen homogeneity within tumors and across tumors sharing the same mutational spectrum. This leads us to raise the concept of “epigenetic addiction” that will be further elaborated herein using pediatric high-grade astrocytomas and a subgroup of embryonal brain tumors as examples.

## High-grade pediatric and young adult astrocytomas: an epigenetic defect of the developing brain?

**Fig. 2:**
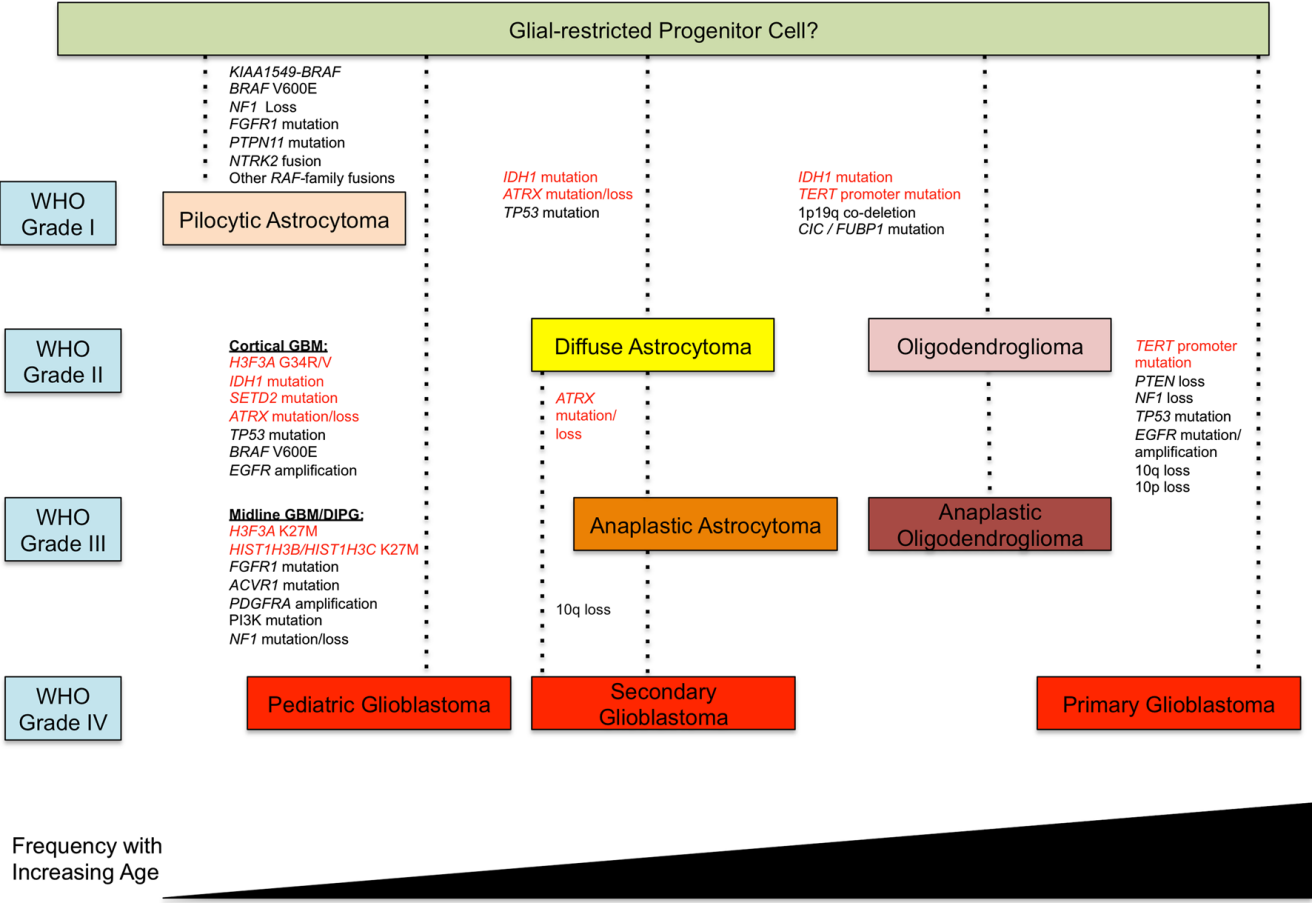
Molecular alterations identified in gliomas across the lifespan. Representation of molecular alterations observed in World Health Organization (WHO) grade I–IV astrocytomas and oligodendrogliomas across the age spectrum. Alterations highlighted in *red text* are shown to have an epigenetic/chromatin remodeling role. *GBM* Glioblastoma, *DIPG* diffuse intronsic pontine glioma

Astrocytomas fall under the larger classification of gliomas, which include ependymomas, oligodendrogliomas and mixed oligoastrocytomas that are more characteristic of the adult years [45]. They are the most common subgroup of brain tumor across age and are themselves comprised of four histological grades, I–II commonly termed low-grade tumors, and III-IV, termed as high-grade tumors [38, 45]. High-grade astrocytomas (HGAs) include grade IV astrocytoma (glioblastoma, GBM) and are particularly lethal and disabling brain neoplasms, with barely 10 % of children and young adults surviving 3 years after their diagnosis. Adult GBM occurring *de novo* (primary GBM) constitutes the large majority of HGA across the lifespan (90 % of all HGA (Fig. 2)). The Cancer Genome Atlas project (TCGA) revealed adult HGA to be highly heterogeneous with numerous mutations and copy number changes within tumors and across tumors sharing similar gene expression profiles [8, 12, 52, 72]. However, in younger patients where a stepwise disease is often identified and leads to secondary GBM, this consortium identified the crucial role for *IDH* metabolic pathways in the genesis of the tumors [7, 12, 52, 54, 72, 78] (Fig. 2). Indeed, recurrent somatic *IDH1* and *2* mutations are found in the vast majority of grade II and III young adult gliomas and secondary GBM [54, 78]. These mutations are extremely rarely present in primary *de novo* GBM [54, 78] and when they do arise, patients are usually young adults aged <25 years [57]). These gain-of-function, heterozygous mutations are initiating events [54, 72, 74] and are associated with two mutually exclusive genetic alterations, *TP53* mutations and 1p19q co-deletions [6, 53] that, respectively, characterize astrocytic and oligodendroglial *IDH*-mutant gliomas. *IDH* mutations induce the production of high quantities of 2-hydroxyglutarate [15]. This onco-metabolite affects chromatin structure through alteration of histone post-translational modifications and global DNA methylation. It competitively inhibits TET-mediated DNA demethylation, resulting in DNA hypermethylation and the glioma-CpG island methylator phenotype (G-CIMP) [52, 68] and impairs histone demethylases [46]. Most strikingly, in children, studies by our group and others have uncovered recurrent mutations directly affecting histone 3 variants at critical residues in pediatric HGA [63, 75]. These studies were the first to identify mutations in regulatory histones to be directly associated with human disease. These mutations were observed in histone 3 genes encoding both non-canonical histone (H3.3) and canonical (H3.1) variants. Interestingly, analogous p.Lys27 Met (K27M) mutations were observed in both of these two variants, with mutually exclusive p.Gly34Arg/Val (G34R/V) seen only in H3.3 thus far [63, 75]. These mutations are the pediatric counterpart of the recurrent *IDH* mutations [54, 78], with which they are also mutually exclusive. Similar to *IDH*, they require association with additional mutations in a specific set of genes that will differ based on the mutated histone variant, age of the patient and the brain location to give rise to HGAs in different anatomic compartments [37, 66]. Importantly, mutations leading to aberrant histone post-translational modifications of two H3 marks, Lysine (K) 27 and K36 appear to be central to the biology of high-grade gliomas in two different neuroanatomical compartments. Mutations affecting H3K27 methylation seem specific to tumors of the midline, encompassing brain regions such as the thalamus, the brainstem (pons), spinal cord and cerebellum. Conversely, mutations affecting H3K36 methylation are prevalent in tumors of the cerebral hemispheres and the genes responsible for these alterations, their accompanying partner mutations, as well as what is known of the downstream effects will be discussed herein.

## Defects in H3K27 at the core of midline and hindbrain tumorigenesis

**Fig. 3:**
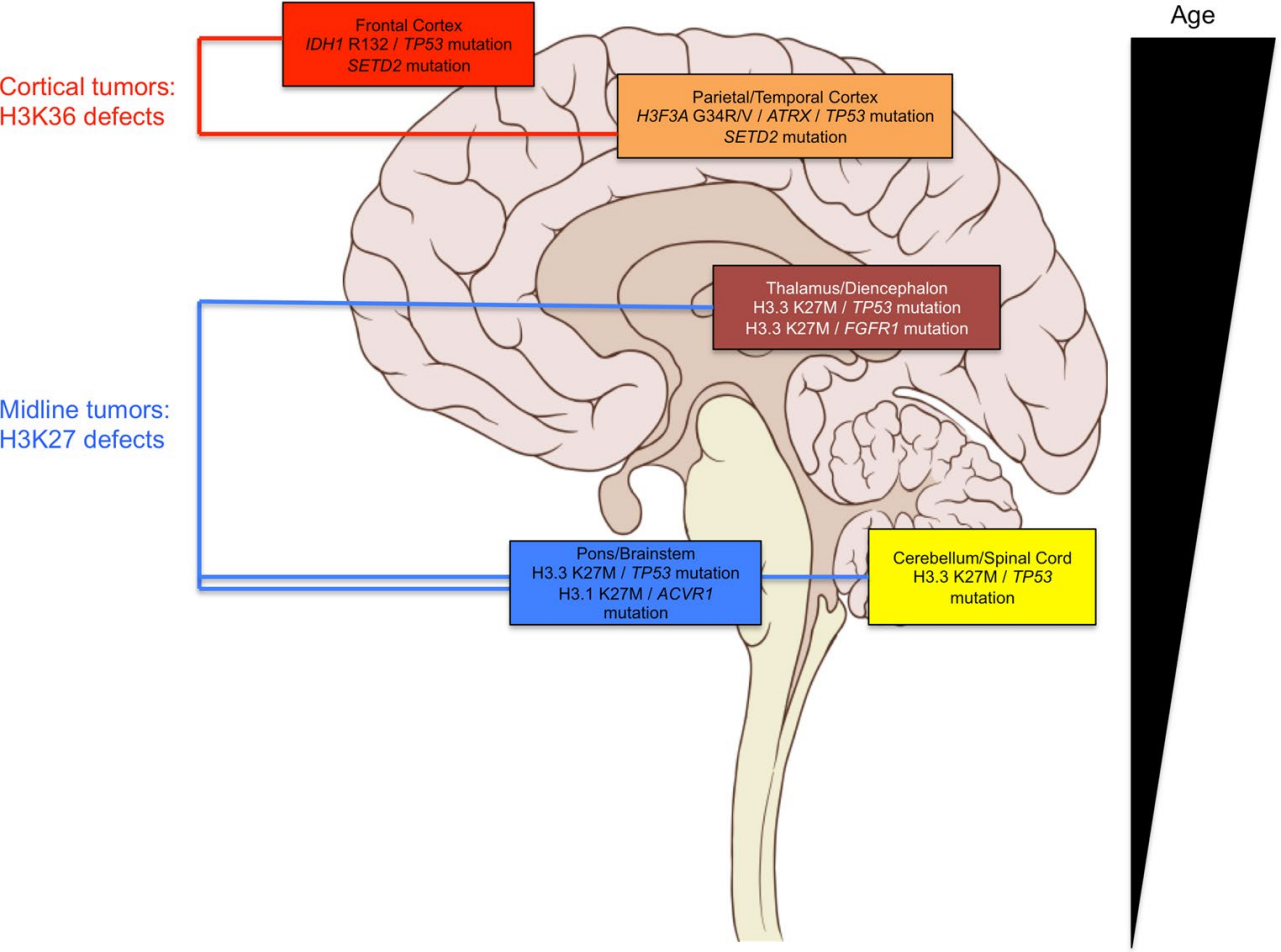
Molecular subgroups of pediatric high-grade gliomas show neuroanatomical preferences. Schematic representation of a sagittal view of the human brain depicting neuroanatomical areas with observed alterations discussed herein. Age of patients harboring these alterations is represented at the right

**Fig. 4:**
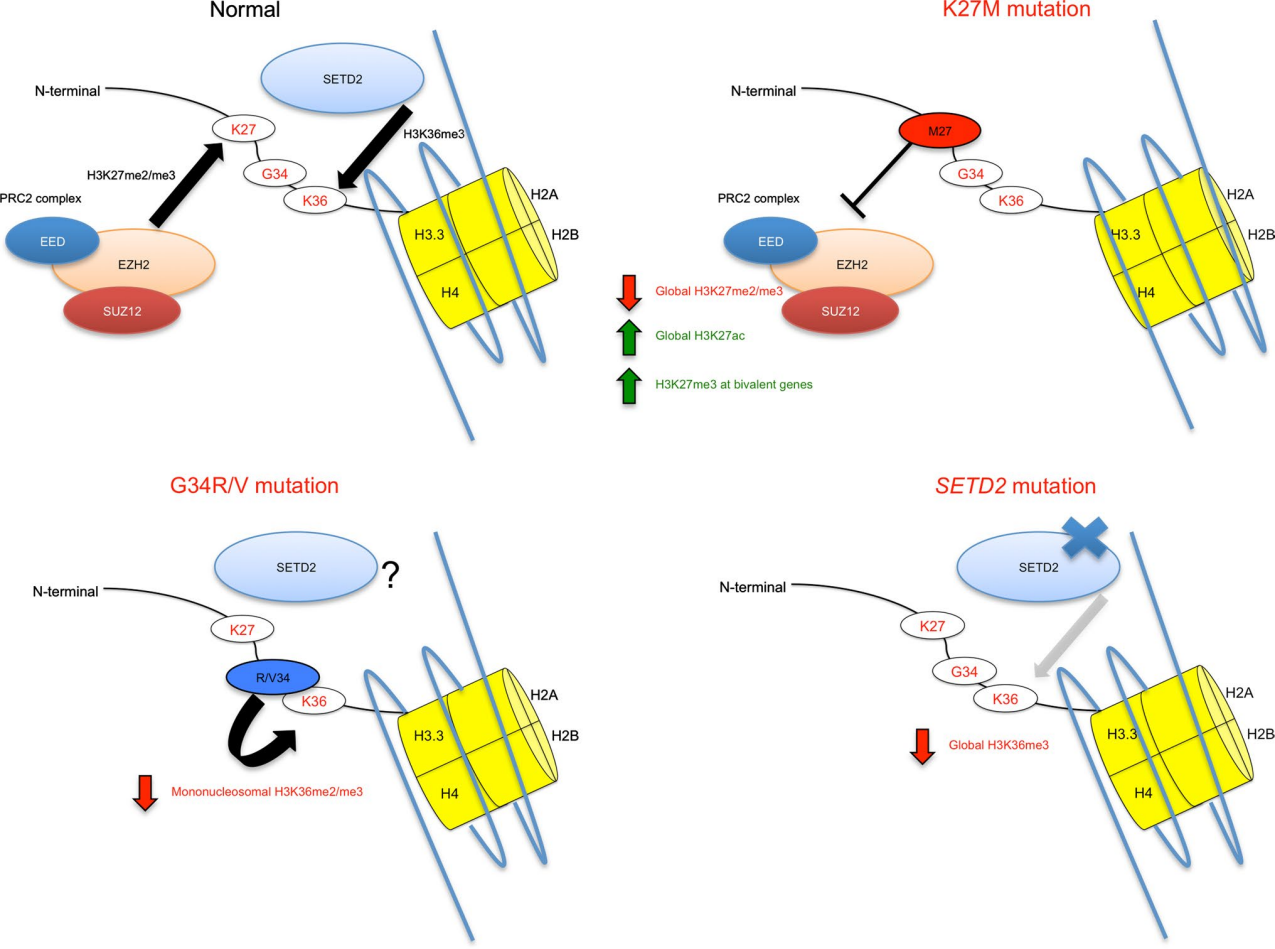
Mutations in H3 and the epigenetic machinery in pediatric high-grade gliomas. Graphic representation of histones containing H3.1, encoded by *HIST1H3B* or *HIST1H3C,* and H3.3, encoded by *H3F3A*, variants and mutations affecting these variants in midline and cortical pediatric high-grade gliomas. Effects of these mutations on histone post-translational modifications are indicated

NGS studies have uncovered a prevalence of H3.3 and H3.1 K27M mutations in HGA tumors arising in regions along the neuroanatomical midline and hindbrain [37, 63, 66, 75]. These include diffuse intrinsic pontine glioma (DIPG, HGA occurring in the pons), cerebellar, spinal and thalamic HGA, which are notoriously difficult areas for surgical resection and stereotactic biopsy. Recently, four concurrent studies helped further shape the genomic landscape of midline HGA [9, 19, 67, 76]. K27M mutations arising in genes encoding the canonical histone H3.1 (*HIST1H3B* or *HIST1H3C*) account for ~20 % of DIPG, and in a dataset of 40 midline HGA tumors, K27M mutations across H3.3 and H3.1 were present in 93 % of tumors in the midline compartment [19, 75]. H3.1 K27M mutations affect younger children (mean age of 3–5 years) and occur exclusively in the brainstem in conjunction with recurrent gain-of-function somatic mutations in the activin A receptor, type I (*ACVR1*). The mutated amino acid residues in *ACVR1,* which encodes a serine threonine kinase, ALK2, have previously been reported as germline mutations causing fibrodysplasia ossificans progressiva (FOP), an inherited musculoskeletal disease [64]. They result in ligand-independent activation of the kinase leading to increase of bone morphogenetic protein (BMP) signaling and increased phospho-SMAD1/5/8 production in tissues [9, 19, 67, 76]. The lack of reported CNS tumor development in FOP patients or Acvr1/Alk2 mouse models suggests that aberrant activation of this pathway is not sufficient for tumorigenesis [22]. *ACVR1* mutations may act in concert with H3 K27M mutations and other associated alterations identified in the PI3K pathway to induce tumorigenesis [19]. Interestingly, in rare cases, somatic-activating *ACVR1* mutations are identified without association with H3 K27M mutations [76], inferring their importance in tumor formation, even though their exact role remains unknown. K27M in H3.3 occurs mainly in association with somatic loss-of-function genetic *TP53* alterations [19, 63]. They represent the vast majority of pediatric midline HGA in the thalamus (80 %), cerebellum and spine (most tumors) or the pons (60 %) (Fig. 3). The recently uncovered hotspot mutations in the fibroblast growth factor receptor 1 (*FGFR1*), leading to hyperactivity along the MAPK axis identified in PA, are also identified in a rare subset of pediatric HGA of the thalamus and notably seem invariably associated with H3.3 K27M mutations [35]. In addition, DIPGs and HGA from other sites have previously been associated with activation of *PDGFRA* through genomic amplification or activating mutations [55, 79]. The gain-of-function alterations in these three growth factor receptors, *ACVR1*, *FGFR1* and *PDGFRA,* associate with H3 K27M variants in midline HGA and are not seen concurrently. *ACVR1* and *FGFR1* mutations are mutually exclusive with *TP53* alterations and occur in specific locations within the midline of the brain (Fig. 3) [19]. K27M mutations appear to correlate with poorer overall survival [37]. A glimpse of the complexity that lies in exploring H3K27 mutations is suggested by their inherent nature to alter global levels of H3K27me3, even though in the case of H3.3K27M mutations H3.3 is a minimal contributor to total histone H3 levels [43]. Independent studies have reported global loss in H3K27me3 associated with K27M mutations in HGA (Fig. 4) [10, 43, 71]. K27M appears to affect endogenous levels of H3K27me3 in human tumor samples, as well as decrease H3K27me3 levels when expressed ectopically (as H3.3 or H3.1 K27M) in a variety of cell types [10, 43]. Interestingly, in tissue samples, this decrease in H3K27me3 is not associated with differences in the levels of *EZH2* expression [71]. This phenomenon was explored directly by Lewis and colleagues utilizing in vitro histone methyltransferase assays in the presence of synthesized H3K27M peptides that demonstrated that the K27M moiety was a potent inhibitor of EZH2 H3K27 tri- and di-methyltransferase activity [43]. Specific amino acid identities at the K27 position are critical to the ability of mutated H3K27 to block EZH2 activity, with methionine (M27) and isoleucine (I27) substitutions demonstrating potent inhibition of the enzyme, hypothesized to result from hydrophobic interactions with the EZH2 active site mediated by their side chains [43] (reviewed in [48]). Interestingly, although H3K27me3 levels are decreased in the presence of K27M mutations, Chan and colleagues demonstrate that there is a striking increase of H3K27me3 marks in association with sequences also marked by H3K4me3 [10]. These include so-called bivalent genes which are potentially poised for expression following removal of the repressive histone methyl mark at K27 [10]. One possible hypothesis is that these dually marked bivalent genes represent a gene signature specific to K27 mutant tumors. This is supported by their Gene Ontology (GO) analysis which suggests they represent genes with significance of cancer pathways as well as developmental pathways involved in pattern specification and morphogenesis [10]. Future experimentation and modeling of individual genes and pathways aberrantly regulated in K27M-mutant tumors are necessary to determine the ones important in specifying tumorigenesis in midline brain regions, where these mutations arise. Introducing K27M expression specifically to various regions in mice, or in cell models derived from these regions of the brain, such as cortical, brainstem or cerebellar astrocytes or neural stem cells will potentially allow further insight into its oncogenic effect. These studies have been performed in the context of low-grade glioma biology studies of *KIAA1549*-*BRAF* fusion, which arises most commonly in pilocytic astrocytoma tumors of the cerebellum [34]. The introduction of the *KIAA1549*-*BRAF* fusion gene was shown to specifically alter growth in cerebellar neural stem cells [36]. Such effects may not be limited to developing or immature astrocytes or stem cells, as lentiviral introduction of specific oncogenes has been shown to lead to re-programming and tumorigenesis of mature neurons and astrocytes [21]. Results demonstrating the global implication of K27M mutations and aberrant levels and distribution of H3K27me3 marks in pediatric brain tumors in specific regions further accentuate the core of this disease in the childhood years as epigenomic in nature (reviewed in [48, 65]). Of importance, H3K27 methylation alterations do not seem to be limited to tumors of the astrocytic lineage, with pediatric medulloblastoma tumors, which are neuronal in origin and occur in the cerebellum, demonstrating H3K27 post-translational modifications through increased EZH2 expression or loss-of-function mutations in *KDM6A*, which encodes a H3K27me3-specific demethylase [17, 51, 59]. Further to this, recent reports of posterior fossa ependymoma group A (PFA) tumors primarily affecting infants in the lateral cerebellum, demonstrate heavy involvement of epigenetic defects revolving around aberrantly regulated H3K27me3 rather than recurrent SNVs; a very unique finding among cancers sequenced to date [47]. The question then becomes: why do tumors in these midline regions harbor K27M mutations and aberrant H3K27 methylation so frequently? What genes are de-regulated by these alterations preferentially in cells of these areas that may contribute to tumorigenesis? Is aberrant H3K27 methylation, shown to be affected by H3.3 and H3.1 K27M mutations, central to tumorigenesis of these areas? If not what accompanying mutations/alterations are required in certain cases? Further experimentation and modeling of these mutations are absolutely essential to answering these questions.

## Defects affecting H3K36 define a unique set of tumors with a variety of clinical and biologic parameters

G34R/V mutations in *H3F3A* were identified in a subset of pediatric GBM defined by a unique set of clinical parameters, namely an adolescent/young adult age group, cortical brain location and in consistent association with concurrent mutations in *TP53,* similar to *IDH* mutant astrocytomas, and in *ATRX* (α-thalassemia/mental retardation syndrome-X-linked) [63], which encodes a subunit of a chromatin remodeling complex required for H3.3 incorporation at pericentric heterochromatin and telomeres [16, 42], in all cases initially identified [63]. In addition, G34R and G34V peptides were shown to decrease levels of H3K36me2/3 in mononucleosomes by mass spectrometric analyses (Fig. 4) [43]. In similar fashion to findings following ChIP-Seq analysis of H3K27me3 marks in K27M-mutant cells [10], Bjerke et al. [5] demonstrate re-distribution or differential binding of H3K36me3 across the genome in a cell line harboring an H3.3 G34V mutation. Further to this, specific upregulation of *MYCN* is observed in this cell line and is suggested to be mediated through G34-mutant H3K36me3 differential binding [5]. H3K36me3 is affected in tumors mutant for isocitrate dehydrogenase 1 (*IDH1*) indirectly through its newly acquired neomorphic enzyme activity enabling conversion of the normal product of oxidative decarboxylation of isocitrate, alpha-ketoglutarate (alpha-KG), into 2-hydroxyglutarate (2-HG) [15, 46]. This mutation, identified in a large proportion of secondary GBM and low-grade gliomas detailed above [54, 78], inhibits histone demethylases, including those acting on H3K36 [46], as well as alpha-KG-dependent dioxygenases [77], has also been shown to be sufficient for establishment of the G-CIMP [52, 68]. Initially, gene expression analysis demonstrated unique clustering of a group of adult GBM tumors defined by *IDH1* mutation and proneural gene signatures with a better overall survival [72]. In addition, cohorts encompassing pediatric and adult GBM tumor samples demonstrated that *IDH1*-mutant GBMs formed a clinically and biologically distinct subgroup, with tumors largely occurring in cortical regions [66]. Recent conditional knock-in mouse models of *IDH1* mutation recapitulated the aberrant histone marks present in overexpression models in cell culture [61]. Further exploration of whole-exome sequencing (WES) datasets of pediatric high-grade gliomas (HGGs) revealed a statistical enrichment of mutations in the H3K36 trimethyltransferase *SETD2* in the pediatric subset compared to 543 non-cancer control exomes [20]. These mutations were associated with global decreases in H3K36me3 levels in HGGs harboring either missense or truncating mutations in *SETD2*, suggesting loss-of-function (Fig. 4) [20]. Loss-of-function mutations in *SETD2* have been associated with renal cell carcinoma, breast cancer, and further confirmed in larger, independent TCGA GBM datasets [13, 14, 24, 49, 70]. Recently they have also been identified in acute leukemia associated with specific driver fusion genes [80]. In the context of pediatric HGGs this is particularly significant, as for pediatric HGGs located in the cerebral hemispheres, approximately half of tumors demonstrate defects in H3K36 methylation acquired by mutations either in *H3F3A* (G34R/V), *IDH1* or *SETD2* and seem to primarily affect adolescent/young adult patients [20, 66]. Future studies are needed to model H3K36 methylation defects as a specific pathway affected in cortical tumorigenesis where alterations affecting the H3K27 mark are rare.

A recent study identified recurrent H3.3 mutations in the vast majority of chondroblastoma and giant cell tumors of the bone, two tumors affecting soft tissues and younger patients (adolescents and young adults primarily) [3]. *H3F3B *K36M mutations were identified in 68/77 of chrondroblastoma samples and *H3F3A *G34W mutations were identified in 48/53 giant cell tumors of bone (GCT); in addition to cases with rare variants *H3F3A *K36M or G34L in chondroblastoma or GCT respectively [3]. No additional genetic alterations were identified to be associated with these H3.3 mutations that appeared to be the sole drivers of these tumors. To date, these are the only group of tumors other than pediatric HGA where a histone gene is recurrently affected in cancer. They illustrate that the residue and histone 3 isoform targeted is specific to age, tumor type and tumor location.

## Mechanisms for telomere lengthening vary with age and tumor type

ATRX is a critical member of a multiprotein complex that includes DAXX and plays a role in regulating chromatin remodeling, nucleosome assembly, telomere maintenance and deposition of histone H3.3. The H3.3 chaperone HIRA loads H3.3 at active and repressed genes and at several transcription factor binding sites, while the ATRX–DAXX complex mediates H3.3 deposition in silent chromatin at telomeres, where the presence of H3.3 is correlated with the repression of telomeric RNA transcription, and near certain specific active genes [25]. Hypomorphic germline mutations in ATRX lead to the α-thalassemia/mental retardation X-linked syndrome. Conversely, complete loss-of-function mutations have recently been identified in cancers, including pancreatic neuroendocrine tumors (PanNETs), neuroblastoma and alpha-thalassemia myelodysplasia syndrome. We and others showed ATRX to be mutated in pediatric HGA [28, 63] and adult *IDH*-mutant astrocytomas [32, 44] and showed alternative lengthening of telomeres (ALT) to be associated with *ATRX* mutations [28, 63]. These mutations are mutually exclusive with *TERT* promoter mutations responsible for telomere elongation that seem to specify primary GBM and oligodendroglial *IDH*-*CIC/FUBP1* mutants (Fig. 2). Interestingly, younger patients with HGA, mainly patients with DIPG did not harbor *TERT* promoter mutations possibly reflecting age and the effect of the cell of origin [9, 19, 76].

## DNA methylation and its role in pediatric brain tumorigenesis

In addition to histone code alterations observed in the pediatric and young adult form of HGA, studies utilizing high-density methylation arrays have revealed striking associations between histone code alterations and global DNA methylation patterns. Sturm and colleagues identify six subgroups of GBM that vary in both clinical and mutational variables associated with individual methylation subgroups; three of which are delineated by mutations shown to affect the histone code, namely H3.3 K27M, H3.3 G34R/V and *IDH1* R132-mutated tumors [66]. Additional subgroups are comprised largely of adult tumors with classical oncogenic alterations such as *EGFR, PDGFRA* amplification and mesenchymal profiles [66], suggesting that epigenomic dysregulation in the form of histone code alterations may be at the core of pediatric tumorigenesis specifically. Herein we have detailed the mutations and alterations leading to histone methylation defects in pediatric brain tumors, and have indicated that these are in addition, associated with novel DNA methylation patterns. Utilizing an adult glioma dataset, Noushmehr and colleagues [52] were able to subgroup tumors via DNA methylation profiling and identify a prominent G-CIMP subgroup defined by *IDH1* mutation, which was later shown as detailed above, to be sufficient to create this phenotype [68]. Expanding global DNA methylation analyses to incorporate a significant pediatric subset, H3.3 K27M, H3.3 G34R/V and *IDH1* mutant tumors were shown to specifically map three epigenetic subgroups of GBM that were comprised largely of pediatric and young adult tumors [66]. As a technology to classify pediatric brain tumors, DNA methylation profiling constitutes quite a robust method, with recent reports demonstrating this for not only gliomas, but also medulloblastoma, pilocytic astrocytoma, ependymoma and primitive neuroectodermal tumors (PNETs) as well as embryonal tumors with multilayered rosettes (ETMRs) [4, 29, 39, 41, 47, 62]. Although in medulloblastoma, DNA methylation derived subgrouping corresponds very well with subgrouping performed via gene expression microarrays [29], for GBM this has proven more difficult, with only supervised analyses demonstrating unique gene expression associated with the epigenetic subgroups defined by H3.3 K27M and G34R/V [63]. Bender and colleagues demonstrate however that H3K27me3 and DNA hypomethylation areas may lie at the core of gene expression programs driven by K27M mutations [4]. Their data also argue for caution in isogenic cell lines expressing H3.3 mutations, as these may not recapitulate H3K27me3 and DNA methylation profiles present in human tumor tissue [4]. Recent multidimensional studies of DIPG tumors confirmed a global landscape of DNA hypomethylation seen in K27M-mutant DIPG tumors, and showed distinct subgrouping of tumors with activated Hedgehog (Hh) or N-Myc (*MYCN*) seen by corroborative transcriptomic and proteomic studies [60]. Differences in DNA methylation profiles of DIPG tumors associated with *MYCN* activation were first shown as delineating one of three epigenetic subgroups across 28 DIPG samples analyzed by methylation arrays [9]. *MYCN* alterations occurred independently of H3 K27M and *ACVR1* mutations, although only a small sample size of *MYCN-*group tumors (*n* = 2) were included in methylation analysis necessitating future investigation of this *MYCN* subgroup [9]. Boot-strapping assessments of DNA methylation data of DIPG and other pediatric HGA tumors strongly suggest that K27M mutations across H3.3 and H3.1 govern distinct epigenomic profiles [19]. Subgroup-specific modeling of HGA and DIPG associated with these particular alterations presents a challenge and will undoubtedly represent a critical step forward in the study of this multifaceted group of diseases. Accurate recapitulation of the striking DNA methylation signatures we see robustly in tumors will form a driving force for progress in understanding the biology of individual subgroups of HGAs.

A recent example by our group in demonstrating the complex interplay between genomic alterations, DNA methylation and gene expression has resulted from the study of pediatric embryonal tumors (Fig. 1). While high-grade astrocytomas are more rare in children, embryonal brain tumors are very specific to the pediatric years and are rarely, if ever, seen in adults. These are aggressive high-grade malignant tumors and include medulloblastoma (neuronal high-grade neoplasms in the cerebellum), primitive neuroectodermal tumors (PNETs), atypical teratoid/rhabdoid tumors (ATRT) and a newly described variant embryonal tumor with multilayered rosettes (ETMR) [40, 45]. Recent re-classification of many diverse histological entities into ETMRs prompted a view into the molecular characteristics underlying these aggressive tumors of the early pediatric years [40]. DNA methylation profiling revealed a very distinct global profile for a series of ETMRs when compared to a diverse set of gliomas, PNETs and other brain tumors [39]. RNA sequencing revealed a recurrent fusion between *TTYH1* and the *C19MC* microRNA cluster, which is primate-specific [39]. When assessing significant genes up- or down-regulated in ETMRs specifically, a fetal-specific isoform of the *de novo* DNA methyltransferase *DNMT3B* (isoform 1b) was shown to be increased specifically in ETMRs, notably when assessed across a large dataset of a variety of tumors [39]. This isoform is known to be uniquely expressed in early post-conceptional fetal brain and may underlie the unique cellular differentiation of ETMR tumors. Specific members of the *C19MC* cluster were able to upregulate the 1b isoform associated with a decrease in retinoblastoma-like 2 (*RBL2*), a gene which regulates the expression of *DNMT3B* [39]. Taken together, these data point to a fusion between a gene and the *C19MC* cluster driving expression of the microRNA. This leads to subsequent downstream upregulation of a fetal isoform of a DNA methyltransferase, influencing the global epigenomic signature of ETMR tumors. Herein once more, data points to the epigenome as a potential driver for pediatric brain tumorigenesis. Defining the function of these unique methylation patterns across tumor subtypes will be important to understand how alterations in both the histone code and DNA methylation alter the genome in such a way as to directly mediate tumorigenesis, or act as a permissive environment for transformation.

## “Epigenetic addiction” in pediatric high-grade brain tumors

Adult HGA is characterized by intra and inter-tumoral heterogeneity. Strikingly, our studies in pediatric HGA and ETMR unravel a previously unsuspected level of homogeneity within molecular subgroups of these tumors. Indeed, separate biopsies from H3 mutant HGA showed similar mutational profiles (including H3.3 K27M mutation) and close to identical global DNA methylation patterns, with heterogeneity seen largely for copy number variants in growth regulatory genes, such as *PDGFRA* amplification [19]. Moreover, recurrences following complete global resection of ETMR were identical for both these features in the cases where the primary and recurrent samples were available despite high-dose alkylating agents and/or radiation therapy [39]. These results mirror recent findings in *IDH*-mutant glioma where *IDH* mutation is present universally, independent of grade or recurrence, whereas non-*IDH* mutant gliomas showed strikingly different mutational patterns at recurrence including growth regulatory gene mutations such as *BRAF* V600E [33]. Although further experimentation focused on animal and cell modeling and inhibition of growth following blockade H3 K27M in mutant cells is required, these findings suggest that these tumors are “addicted” to specific forms of epigenetic dysregulation. To meet the criteria of oncogene addiction further experimentation is needed; however, if proven correct, this offers an unprecedented therapeutic opportunity unique to pediatric brain tumors contrary to the landscape of complex heterogeneity seen in epithelial and other cancers.

## Summary

Recent work by our lab, other independent groups as well as large consortia including TCGA and the International Cancer Genome Consortium (ICGC) have shown epigenetic defects to be present in a large proportion of pediatric brain tumors. They call for the advent of molecular pathology, a needed change in the WHO classification to integrate data that takes molecular defects into account when classifying/stratifying a brain tumor. As patients continue to consent to participation in sequencing and molecular studies, the research community will be able to continue to explore the novel epigenetic mechanisms at play underlying their formation. Recent discoveries of histone variant mutations at critical residues have placed defects leading to H3 deposition and post-translational modifications at the center of pediatric high-grade gliomagenesis. Future work modeling the impact of these mutations, the potential need for additional hits or associated mutations and the careful interpretation of the interplay between genomic and epigenomic data may allow us to reconcile major mechanisms ongoing in either dimension. Examples such as ETMR tumors support an integrated approach to studying driving forces governing tumorigenesis. With such an approach, we hope to inform clinical trials with biomarker development and drug discovery fueled by molecular subgrouping of this deadly group of pediatric tumors. Uncovering the untapped biology in this young field in pediatric brain tumors may aid in laying the foundation we need to tackle these tumors clinically to improve outcome for patients.
